# Circadian rhythms remain temperature compensated during a Q neuron–induced hibernation-like state in mice

**DOI:** 10.1371/journal.pbio.3003475

**Published:** 2026-04-15

**Authors:** Arisa Hirano, Tohru M. Takahashi, Hiroto Ashitomi, Kazumasa Z. Tanaka, Takeshi Sakurai

**Affiliations:** 1 International Institute for Integrative Sleep Medicine (WPI-IIIS), Tsukuba Institute for Advanced Research (TIAR), University of Tsukuba, Tsukuba, Ibaraki, Japan; 2 Institute of Medicine, University of Tsukuba, Tsukuba, Ibaraki, Japan; 3 Memory Research Unit, Okinawa Institute of Science and Technology Graduate University, Onna, Okinawa, Japan; 4 Life Science Center for Tsukuba Advanced Research Alliance, University of Tsukuba, Tsukuba, Ibaraki, Japan; Charité - Universitätsmedizin Berlin, GERMANY

## Abstract

The circadian clock is an internal timekeeping system that enables organisms to adapt to daily environmental changes. A defining property of this clock is temperature compensation, whereby the circadian period remains relatively constant despite fluctuations in temperature. Although this phenomenon has been extensively studied in cultured cells and tissues, how the mammalian circadian clock responds to hypothermia in vivo remains largely unknown. Here, we examined circadian dynamics in a hibernation-like state in mice, termed Q neuron–induced hypometabolic and hypothermic state (QIH), which lowers core and brain temperatures to approximately 25 °C for extended periods. We found that free-running behavioral and body temperature rhythms were preserved after QIH, exhibiting only minor phase changes. In vivo recordings further revealed that neuronal firing rhythms in the suprachiasmatic nucleus (SCN) and molecular rhythms of PER2::Luc bioluminescence in peripheral tissues persisted during QIH with dampened amplitudes but largely unaltered circadian periods. In contrast, SCN and kidney slice cultures maintained at the same temperature displayed strongly attenuated or reset PER2::Luc oscillations. Together, these findings demonstrate that the circadian period is robustly temperature compensated in vivo, likely supported by systemic regulatory mechanisms beyond cell-autonomous clockwork. Our results provide new insight into the fundamental biology of circadian robustness and establish a framework for understanding clock function during hibernation and potential medical hypothermia.

## Abstract 2

The circadian clock maintains a nearly constant period across temperatures, but to what extent temperature compensation operates in mammals during deep hypothermia is unknown. This study shows that mice maintain circadian rhythms during a hibernation-like hypothermic state, and that this is supported by systemic regulatory mechanisms.

## Introduction

The circadian clock governs physiological rhythms with a ~24-h period to adapt to environmental changes [1]. In mammals, the central clock resides in the suprachiasmatic nucleus (SCN) of the hypothalamus and orchestrates daily behavioral and physiological rhythms [2,3]. This master pacemaker also synchronizes peripheral clocks located throughout the body, thereby coordinating local tissue functions. Circadian clocks are self-sustained and can generate rhythms even without environmental cues (zeitgebers), while they can also be entrained by external signals such as the light-dark cycle. Another hallmark of the circadian clock is temperature compensation, whereby the circadian period remains largely unchanged despite temperature fluctuations [4,5]. Even though the molecular clock is composed of multiple biochemical reactions (e.g., transcription, translation, and protein modifications), the circadian period is not significantly affected by changes in temperature [6,7]. Temperature compensation is an evolutionarily conserved phenomenon [8–11]. This property is thought to be essential not only for organisms exposed to fluctuating ambient temperatures but also for endothermic animals, for which stable timekeeping supports seasonal adaptation, sleep–wake regulation, and survival strategies such as hibernation. Moreover, understanding clock stability under hypothermia may be of clinical importance, for example, in the context of therapeutic or surgical hypothermia.

In cyanobacteria, a decrease in culture temperature results in dampened circadian oscillation, while the period remains in a similar range. The oscillation stops when the temperature falls below a threshold [12]. Similarly, the mammalian clock becomes damped at low temperature to compensate for the circadian period in vitro and in the mathematical model [6,13]. Thus, a decrease in oscillation amplitude is regarded as a mechanism of temperature compensation, while the precise molecular basis remains incompletely understood. In mammals, phosphorylation of PERIOD (PER) proteins by casein kinase I has been proposed as one mechanism for temperature compensation [14–16]. Post-transcriptional regulation (e.g., alternative splicing and polyadenylation) and Ca^2+^ signaling are also known to be involved in maintaining the period at low temperature [17,18].

Since homeothermic animals such as mammals maintain their body temperature within a narrow range by homeostatic regulation, it is challenging to investigate the effect of temperature changes on circadian oscillation in living animals. Therefore, most studies on temperature compensation in the mammalian clock have been conducted using cell or tissue culture under mild cold exposure. The effect of anesthesia-induced hypothermia on behavioral rhythms has been assessed in hamsters and rats, suggesting interspecies differences [19,20]. However, it is necessary to consider the effect of anesthesia rather than hypothermia itself. Hibernating animals provide another approach, but their body temperature often drops below 10 °C, leading to severe suppression of molecular rhythms [21–23]. Thus, how the mammalian circadian clock behaves under mild hypothermia in vivo remains unclear. Importantly, in vitro systems lack systemic inputs—such as endocrine and autonomic regulation—that may stabilize clock function at the organismal level.

We recently developed a method to manipulate the mammalian body temperature for extended periods by excitation of pyroglutamylated RFamide peptide (QRFP)-producing neurons in the antero-ventral periventricular nucleus (AVPe) of the hypothalamus [24,25]. This population, known as Q neurons, triggers a hibernation-like state (Q neuron–induced hypothermic/hypometabolic state; QIH) in mice, which do not naturally hibernate. QIH provides a unique opportunity to examine clock dynamics in a controlled hypothermic state in vivo. Here, we report that circadian oscillations in behavior, neuronal activity, and molecular rhythms persist during QIH, with preserved period but reduced amplitude, revealing that the circadian clock is robustly temperature compensated in its period in vivo.

## Results

### Effect of QIH on the circadian phase of behavioral rhythms

**Fig 1:**
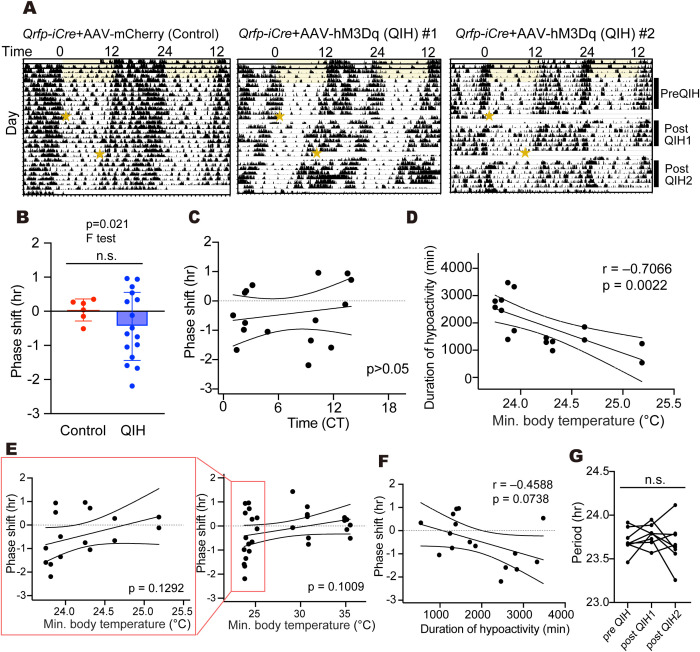
Phase shift of locomotor activity rhythms in QIH. **(A)** Actograms of locomotor activity for mice in the QIH condition. After the entrainment to light-dark (LD) cycle, mice were kept in the constant darkness (DD). The timing of CNO injection was presented as a star in the actogram. **(B)** Phase shift of the locomotor activity rhythms between the pre-QIH and post-QIH period. When the mice showed insufficient QIH (QIH_>27 °C_: min. body temperature of above 27 °C), the sample was excluded from analysis. Data were shown as means with individual plots and SD (Control: *n* = 6, QIH_≦27 °C_: *n* = 16, Control vs. QIH_≦27 °C_: *p* = 0.1045 by Welch’s *t* test, *p* = 0.021 by F test). A positive value indicates phase advance. **(C)** Correlation between the phase shift and the circadian time (CT) at the CNO administration (QIH_≦27 °C_: *n* = 16, p = 0.5026, r = 0.1809 by Pearson’s correlation). **(D)** Correlation between the minimum body temperature and duration of hypoactive state (QIH: *n* = 16, *p* = 0.0022, r = –0.7066 by Pearson’s correlation). **(E)** Correlation between the phase shift and the min. temperature after the CNO administration. The right graph contains data from all QIH mice expressing hM3Dq-mCherry regardless of the degree of minimum body temperature (QIH_≦27 °C_: *n* = 16, QIH_>27 °C_: *n* = 8) and control mice expressing mCherry (*n* = 6, *p* = 0.1009, r = 0.3053 by Pearson’s correlation). The left panel showed the plot for only QIH_≦27 °C_ mice (*n* = 16, *p* = 0.1292, r = 0.3957 by Pearson’s correlation). **(F)** Correlation between the phase shift and duration of QIH (QIH_≦27 °C_: *n* = 16, *p* = 0.0738, r = –0.4588 by Pearson’s correlation). **(G)** The changes in circadian period after the QIH. The period was determined by fitting a line to the activity onsets for pre-QIH (7 days), post-QIH1(6 days), and post-QIH2 (6 days). QIH_≦27 °C_: *n* = 16, *p* = 0.3948 by one-way ANOVA. The period of pre-QIH (7 days), post-QIH1(6 days), and post-QIH2 used for the period calculation were presented in the right panel of Fig 1A. The data underlying this Figure can be found in S1 Data.

We first examined the effect of QIH on the phase of behavioral rhythms, as behavioral rhythms reflect the central clock (SCN) function. If the clock oscillation stops during the QIH and resumes after the arousal (rewarming) from QIH, the phase would be dependent on the timing of recovery. In another case, where the circadian oscillation speed is temperature-sensitive, a large phase shift would be expected. Before the behavioral assessment, *Qrfp-iCre* mice were subjected to stereotaxic injection of an AAV vector Cre-dependently expressing excitatory Designer Receptors Exclusively Activated by Designer Drugs (DREADD), hM3Dq fused with mCherry, in the AVPe for specific manipulation of Q neurons (S1 Fig). Mouse locomotor behavior in the home cage was then continuously monitored with infrared sensors. Under constant darkness (DD), the mouse exhibited free-running behavioral rhythms with an internal circadian period slightly different from 24 h. QIH was induced by administering the DREADD ligand, clozapine-N-oxide (CNO). The phase of activity onset was determined for each day, and the phase difference between pre-QIH and post-QIH was calculated. As expected, control mice showed no obvious activity reduction or phase shift following the CNO injection (Fig 1A and 1B). In contrast, QIH mice showed a pause in activity and recovered from the hypoactive state a few days after the induction. The phase was not significantly changed in the QIH group compared to the control group on average (Fig 1B, Control: *n* = 6, QIH_≦27 °C_: *n* = 16, *p* = 0.1045 by Welch’s *t* test). On the other hand, we found that the QIH group showed a significantly larger variation among the samples compared to the control, suggesting that the circadian phase or oscillation speed (circadian period) possibly became unstable during the QIH (Fig 1B, *p* = 0.021 by F test). Importantly, the phase changes did not depend on the circadian time (CT) at the QIH induction (Fig 1C, QIH_≦27 °C_: *n* = 16, *p* = 0.5026, r = 0.1809, by Pearson’s correlation). Because the duration of hypoactive state was highly correlated with the minimum body temperature (Fig 1D, QIH_≦27 °C_: *n* = 16, *p* = 0.0022, r = –0.7066, by Pearson’s correlation), we also examined the dependency of phase shift on the depth of QIH. There are small tendency of phase delay observed in mice with lower minimum body temperature (Fig 1E, Control: *n* = 6, QIH_>27 °C_: *n* = 8, QIH_≦27 °C_: *n* = 16, *p* = 0.1009, r = 0.3053 by Pearson’s correlation) and longer duration of hypoactive state (Fig 1F, QIH: *n* = 16, *p* = 0.0738, r = –0.4588 by Pearson’s correlation), although they did not reach the statistical significance. Even though the timing of recovery was variable among mice (1,000 ~ 3,000 min after the CNO administration, Fig 1D), probably due to the individual differences in viral expression, the phase shift was restricted within ~1 h advance to ~2 h delay, suggesting that the clock in the SCN kept the circadian period in a similar range during the QIH. Notably, the circadian rhythms of locomotor activity returned normally within a few days with a period unchanged in DD (Fig 1A and 1G, *p* = 0.3948 by one-way ANOVA), indicating that the external zeitgeber cue is not required to resume the rhythmic activity pattern after the arousal from QIH.

We then evaluated the mouse wheel-running activity because the onset of the active period is much clearer in wheel-running than in locomotor activity. Importantly, similar results were obtained for wheel-running activity rhythms. QIH was induced at various time points in DD, and the phase shift of wheel-running activity rhythms caused by QIH was evaluated (S2A Fig). While an averaged phase shift in QIH group was not significantly different from that of the controls (S2B Fig, Control: *n* = 17, QIH_≦27 °C_: *n* = 20, *p* = 0.0859 by Welch’s *t* test), the individual variation was larger in QIH mice (Control *n* = 17, QIH_≦27 °C_ *n* = 20, *p* = 0.0011 by F test), similar to the locomotor activity rhythms. There was no correlation between the induction time and phase changes after the QIH (S2C Fig, QIH_≦27 °C_; *n* = 20, *p* = 0.4561, r = –0.1767 by Pearson’s correlation). We observed a significant correlation between the minimum body temperatures and phase shift, and there is a tendency of phase advance in deeper QIH, while these effects were limited (S2D Fig, Control: *n* = 17, QIH_≦27 °C_: *n* = 20, QIH_>27 °C_: *n* = 12, *p* = 0.0352 r = –0.3016 by Pearson’s correlation). Again, the activity rhythms returned to normal within a few days of recovery from the hypoactive state in DD without entrainment cues (S2A Fig).

### Phase change of body temperature rhythms in QIH

**Fig 2:**
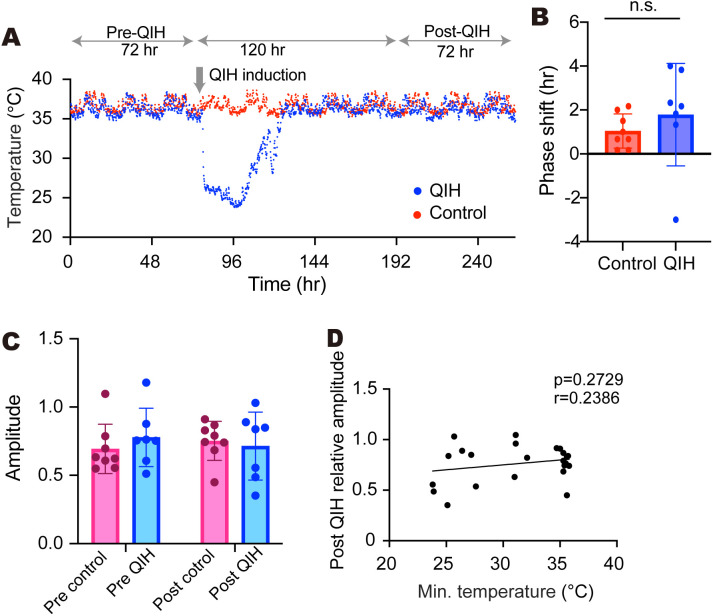
Phase shift of body temperature rhythms in QIH. **(A)** The core body temperature rhythms in QIH mice and control mice. **(B)** The phase shift of core body temperature rhythms after QIH. Core body temperature for 72 h before QIH or 72 h after recovery of QIH was used for the calculation. When the mice showed insufficient QIH (QIH_>27 °C_: min. body temperature of above 27 °C), the sample was excluded from analysis. Data are shown as means with SD (Control: *n* = 8, QIH_≦27 °C_: *n* = 7, *p* = 0.4463 by Welch’s *t t*est, *p* = 0.0103 by F test). **(C)** The amplitude of core body temperature rhythms. Data are shown as means with SD (Control: *n* = 8, QIH_≦27 °C_: *n* = 7, pre vs. post: *p* = 0.9747, Con vs. QIH, *p* = 0.7493, interaction: *p* = 0.4056 by two-way ANOVA). **(D)** Correlation between the rhythm amplitude in the 72 h post-QIH and the minimum body temperature during the QIH. It includes data from all QIH mice expressing hM3Dq-mCherry regardless of the degree of minimum body temperature (QIH_≦27 °C_: *n* = 7, QIH_>27 °C_: *n* = 8) and control mice expressing mCherry (Control: *n* = 8). *p* = 0.2729, r = 0.2386 by Pearson’s correlation. The data underlying this Figure can be found in S1 Data.

The body temperature is under the control of the central clock and exhibits a circadian rhythm correlated with activity. We next compared the body temperature rhythms between pre-QIH (3 days before the CNO induction) and post-QIH (5 days after the recovery from QIH) under DD conditions. During QIH, the temperature did not show a circadian pattern, while the rhythmic pattern reappeared immediately after recovery from hypothermia in DD (Fig 2A). There were no significant changes in the phase of body temperature rhythms between the control and QIH mice (Fig 2B, Control: *n* = 8, QIH_≦27 °C_: *n* = 7, *p* = 0.4463 by Welch’s *t* test). Again, we observed larger variability in the phase shift in the QIH group, similar to the locomotor activity and wheel-running activity analysis (Fig 2B, Control: *n* = 8, QIH_≦27 °C_: *n* = 7, *p* = 0.0103 by F test). Even after the long-term hypothermia, the amplitude of body temperature in post-QIH was equivalent to that of the control group and that in pre-QIH, even in the absence of entrainment cues (Fig 2C, Control: *n* = 8, QIH_≦27 °C_: *n* = 7, pre versus post: *p* = 0.9747, Con versus QIH, *p* = 0.7493, interaction: *p* = 0.4056 by two-way ANOVA). We also found no correlation between the minimum temperature in QIH and the amplitude of body temperature rhythm after the QIH (Fig 2D, Control: *n* = 8, QIH_≦27 °C_: *n* = 7, QIH_>27 °C_: *n* = 8, *p* = 0.2729, r = 0.2386 by Pearson’s correlation). Taken together with the activity rhythm results, these results indicate that the circadian clock maintains a period similar to that observed under normal conditions, even during QIH.

### Circadian oscillation of SCN neurons during QIH

**Fig 3:**
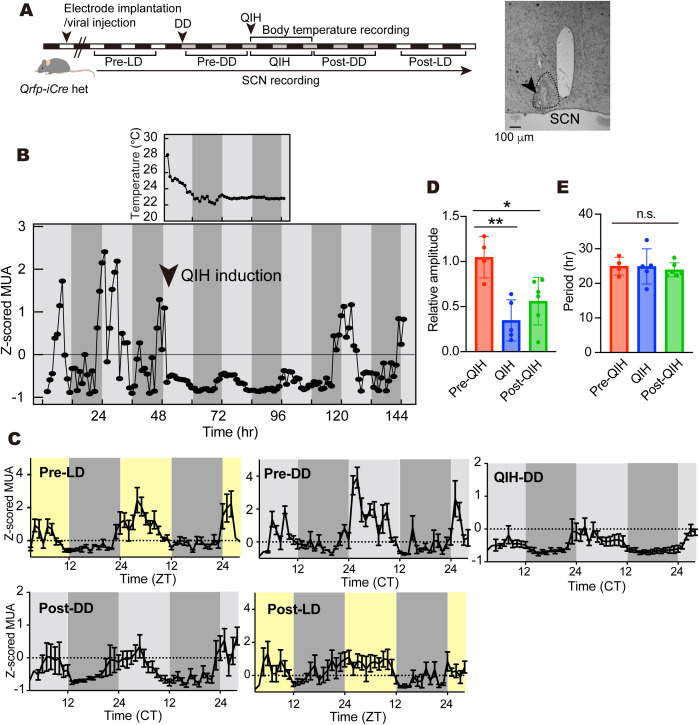
Effect of QIH on SCN neuronal activity rhythms. **(A)** Experimental design of single-unit activity recording in SCN neurons under the QIH condition. **(B)** The representative trace of neuronal activity and body temperature for 48 h in each condition (pre-QIH, QIH, and post-QIH in DD). The body temperature was recorded by a thermal camera during the QIH. **(C)** Standardized data are shown as means with SD. Data were independently obtained from 6 mice and data with technical trouble were excluded (pre-QIH in LD: *n* = 5, pre-QIH in DD: *n* = 4, QIH: *n* = 5, post-QIH in DD: *n* = 6, post-QIH in LD: *n* = 5). **(D)** The amplitude of neuronal activity rhythms in each condition in DD. Data are shown as means with SD (pre-QIH: *n* = 4, QIH: *n* = 5, post-QIH: *n* = 6, *: *p* < 0.05 and **: *p* < 0.01by one-way ANOVA followed by Tukey’s test as a post-hoc test). **(E)** The period of neuronal activity rhythms in each condition in DD. Data are shown as means with SD (pre-QIH: *n* = 4, QIH: *n* = 5, post-QIH: *n* = 6, *p* > 0.05 by one-way ANOVA). The data underlying this Figure can be found in S1 Data.

Given that the circadian phases of behavioral and body temperature rhythms were not largely affected by QIH lasting a few days, we assumed that the circadian clock continued oscillating during the QIH. Thus, we then examined the firing rhythms of SCN neurons at a single neuronal level by in vivo electrophysiology under DD conditions (Fig 3A). We observed high neuronal activity in the subjective daytime and low activity during the subjective night in both LD and DD conditions before QIH induction (Fig 3B, a representative trace of neuronal activity and Fig 3C, averaged activity pattern from multiple mice), which was consistent with the previous study [26]. We then induced QIH in DD. Although the induction of QIH significantly reduced the firing rate of the SCN neurons, a circadian fluctuation with a small amplitude was clearly observed in QIH mice (Fig 3B). During the recording, the body temperature was maintained below 24 °C (Fig 3B, top panel). We observed a small change in the phase of rhythms after QIH (Fig 3C, post-QIH), likely reflecting the large variation among individuals observed in activity rhythms and body temperature rhythms. Using the 48-h data in each condition, we calculated the rhythm amplitude and period by fitting a sine curve to the normalized data. The amplitude of the SCN firing activity was significantly reduced in QIH, and this effect persisted even after the recovery from the hypothermia (post-QIH) (Fig 3D, pre-QIH: *n* = 4, QIH: *n* = 5, post-QIH: *n* = 6, Pre-QIH versus QIH; *p* = 0.0028, Pre-QIH versus Post-QIH; *p* = 0.0231 by Tukey’s test). In contrast, the circadian period during QIH was not significantly changed compared to the pre-QIH and post-QIH (Fig 3E), indicating that the circadian period of the SCN neuronal activity rhythms is also well temperature compensated in mice with low body temperature.

Our electrophysiological data clarified that the SCN continues to oscillate during QIH. However, the oscillation might be maintained because the SCN temperature did not decrease, possibly due to the local brain thermogenesis. To exclude this possibility, we recorded the brain temperature surrounding the SCN using a thermocouple probe placed above the SCN (S2A, S2B Fig). We successfully achieved long-term recordings of brain temperature at high temporal resolution (2 Hz) in freely moving mice during QIH (S2C Fig). The SCN temperature was highly correlated with core body temperature, which was simultaneously measured using an intraperitoneally implanted thermo-sensor (S2D Fig). During the induction phase of QIH, there was a slight difference between core body and brain temperatures, but this difference diminished to nearly zero during stable QIH (4 h after the induction, S2E, S2F Fig). Since we confirmed that the temperature in the brain dropped to ~25 °C during the QIH, we used core body temperature as a proxy for SCN temperature in subsequent experiments.

### In vivo PER2 rhythms in QIH

**Fig 4:**
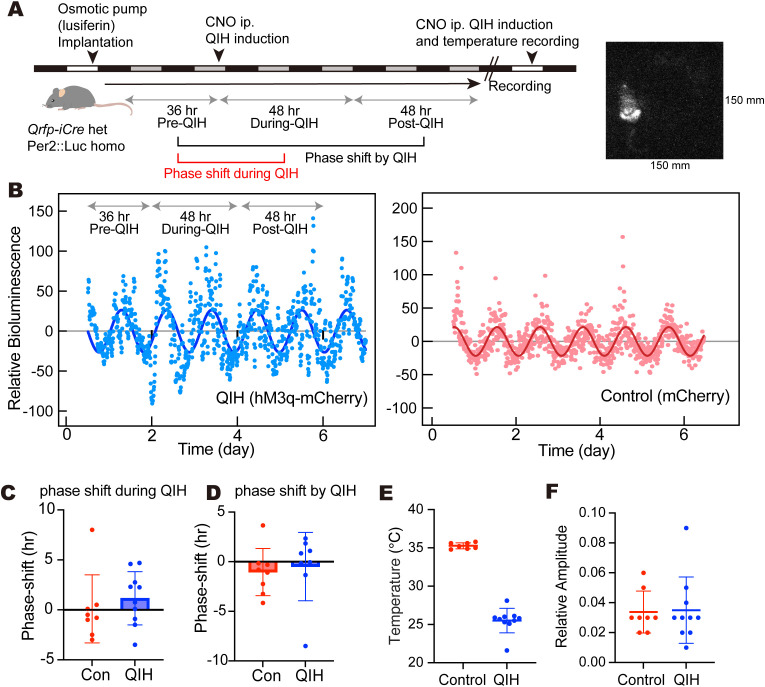
PER2::LUC rhythms in QIH mice. **(A)** Experimental design of in vivo PER2::LUC recording under the QIH condition and representative image of bioluminescence. Images were taken every 10 min, and the exposure time was 5 s for each time. **(B)** The representative bioluminescence traces from QIH mice (left) and control (right). Bioluminescence intensity was detrended and plotted. Two days after the recording started, CNO was administered to induce QIH. Data from all animals are found in S1 Data. **(C)** The phase shift of bioluminescence rhythms between pre-QIH (day 1–2) and QIH (day 3–4). Data were shown as means with individual plots and SD, and no significant difference was observed (Control: *n* = 8, QIH: *n* = 10, *p* = 0.4855 by unpaired *t* test). **(E)** The phase shift of bioluminescence rhythms between pre-QIH (day 1–2) and post-QIH (day 5–6). Data were shown as means with individual plots and SD, and no significant difference was observed (Control: *n* = 8, QIH: *n* = 8, *p* = 0.7132 by unpaired *t* test). **(F)** The confirmation of body temperature after the induction of QIH. The minimum body temperature was plotted (Control: *n* = 7, QIH: *n* = 10). Data were shown as means with individual plots and SD. **(G)** The amplitude of bioluminescence rhythms during the QIH (Control: *n* = 8, QIH: *n* = 10, *p* = 0.892 by unpaired *t* test). The data underlying this Figure can be found in S1 Data.

Since peripheral organs are subjected to cold exposure during QIH, we also investigated the effect of QIH on peripheral clocks. In normal conditions, the peripheral clocks receive the neuronal and endocrine inputs from the SCN clock for synchronization. To monitor the molecular rhythms in vivo, we utilized PER2::LUC knock-in mice, in which the *Luciferase* was inserted in the *Period2* gene, one of the core clock genes [27]. We continuously infused luciferin into mice via an intraperitoneally implanted osmotic pump and recorded the bioluminescence under DD conditions (Fig 4A). Since PER2 is ubiquitously expressed, the bioluminescence was derived from multiple tissues, while it is assumed that tissues located on the surface with high PER2 expression mainly contribute to the signals [28]. Forty-eight hours after the start of recording, we injected CNO to induce QIH and continued recording. A clear bioluminescence oscillation of PER2::LUC was observed in both the control mice expressing mCherry and QIH mice expressing hM3Dq-mCherry in Q neurons (Fig 4B). The phase shift of PER2 rhythms between pre-QIH (day 1–2) and during QIH (day 3–4) was not detected in the QIH group (Fig 4C, Control: *n* = 8, QIH: *n* = 10, *p* = 0,4,855 by unpaired *t* test). Similarly, the phase shift of bioluminescence rhythms between pre-QIH (day 2) and post-QIH (day 5–6) did not occur (Fig 4D, Control: *n* = 8, QIH: *n* = 8, *p* = 0.7132 by unpaired *t* test), suggesting that reduction of temperature, low temperature, and rewarming did not significantly affect the PER2 rhythms. We confirmed mice of the QIH group showed a sufficient reduction of body temperature (Fig 4E). The amplitude of PER2::LUC signal rhythms during the two days after the CNO injection was not affected by QIH (Fig 4F, Control: *n* = 8, QIH: *n* = 10, *p* = 0.892 by unpaired *t* test), although the temperature-dependency in bioluminescence signals should be carefully considered (e.g., enzymatic activity, metabolism of luciferin, and production of ATP). These findings suggest that circadian oscillation in the peripheral tissues also persisted during the QIH and there is minimal effect on its phase and circadian period, confirming robust temperature compensation in peripheral molecular clocks.

### In vitro SCN culture in cold environment

**Fig 5:**
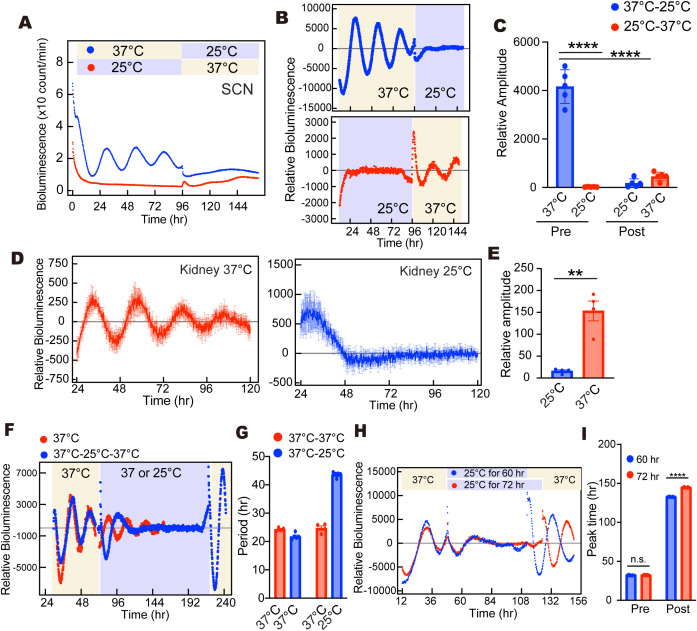
Circadian rhythms in cultured slices. **(A)** Recording of PER2::LUC bioluminescence rhythms in in vitro SCN slice at low culture temperature. Raw representative trace of bioluminescence was plotted. Data from all animals are found in S1 Data. **(B)** The detrended bioluminescence rhythms. **(C)** The amplitude of detrended PER2::LUC bioluminescence rhythms in the SCN slice culture (37–25 °C: *n* = 5, 25–37 °C: *n* = 5, ****: *p* < 0.0001 by Fisher’s LSD). Data were shown as means with individual plots and SD. **(D)** Recording of PER2::LUC bioluminescence rhythms in in vitro kidney slices at low culture temperature. The detrended bioluminescence rhythms were shown. **(E)** The amplitude of detrended PER2::LUC bioluminescence rhythms (37 °C: *n* = 4, 25 °C: *n* = 4, *p* = 0.0084 by Welch’s *t* test, *p* = 0.0057 by F test). Data were shown as means with individual plots and SEM. **(F)** The detrended PER2::LUC bioluminescence rhythms in the kidney slice. Data from all slices are found in S1 Data. **(G)** The period of bioluminescence rhythms shown in (F) (37 °C: *n* = 4, 37 to 25 to 37 °C: *n* = 5). Data were shown as means with SEM. **(H)** The effect of timing of rewarming the culture media on the circadian phase in the kidney slices. The detrended PER2::LUC bioluminescence rhythms were plotted. Data from all slices are found in S1 Data. **(I)** The peak time of PER2 bioluminescence was calculated from the rhythm data without detrending treatment. (60 h: *n* = 5, 72 h: *n* = 5, ****: *p* < 0.0001 by Fisher’s LSD). Data were shown as means with SEM. The data underlying this Figure can be found in S1 Data.

Previous studies demonstrated that mammalian molecular rhythms (e.g., *Per2*-*Luc* and *Bmal1*-*Luc*) were significantly dampened in cold in vitro culture conditions [18,29]. We first confirmed the circadian oscillation of PER2::LUC in the SCN slice culture prepared from adult mice at 25 °C, which is equivalent to the brain temperature in the QIH state (Fig 5A and 5B). Although the PER2::LUC rhythm was clearly observed at 37 °C, low culture temperature attenuated the rhythmic PER2 expression (Fig 5C). Importantly, when we returned the culture temperature to 37 °C, the PER2::LUC rhythm was partially restored, suggesting that a part of SCN neurons remained viable at 25 °C. This experiment indicated the sharp contrast between the dynamics of the central circadian clock in the SCN in vivo (Figs 1–3) and that in vitro (Fig 5).

### Cold exposure resets the kidney clock in vitro

We similarly found distinct responses of peripheral rhythms to cold/hypothermic conditions between in vivo and in vitro experiments. We prepared kidney slices from PER2::LUC knock-in mice, and continuously recorded the cellular rhythms of PER2::LUC bioluminescence. Culture at the low temperature (25 °C) generated a large initial fluctuation of bioluminescence. However, it was transient, and not a rhythmic pattern, and the amplitude of PER2::LUC rhythm was significantly reduced (Fig 5D and 5E). We confirmed cell viability at 25 °C by rewarming the cultured cells (37 °C to 25 °C to 37 °C, Fig 5F). Again, culture at 25 °C resulted in a large fluctuation of the bioluminescence followed by a flattened PER2::LUC rhythm. Importantly, the period calculated by sine curve firing exceeded 40 h at 25 °C, which was no longer circadian (Fig 5F and 5G). When the culture temperature returned to 37 °C (25–37 °C), the circadian oscillation was rescued, indicating that low temperature culture did not compromise cell viability (Fig 5F). We also noticed that the increase in culture temperature strongly reset the circadian clock in kidney slice cultures. Accordingly, large phase changes depending on the timing of temperature elevation (25–37 °C) were observed (Fig 5H). In contrast, this phase shift, which depends on the timing of recovery from low temperature (corresponding to hypothermia in vivo), was not observed during QIH (Figs 1 and 4). Thus, we conclude that the circadian clock continues oscillating during QIH (~25 °C) and its period is resistant to the cold environment in vivo, while PER2::LUC activity in tissue cultures failed to maintain oscillation and circadian period.

## Discussion

In the present study, we evaluated the behavior of the circadian clock in the induced hibernation-like state (QIH, [24]) in mice. Real-time recording of neuronal activity revealed that the circadian clock continues to oscillate in this state, even with significantly reduced amplitudes. Although we observed a tendency of phase delay of locomotor rhythms after the long-term QIH, the change was less than 1.5 h, suggesting that the circadian period was well temperature compensated. However, the variation among individuals was significantly larger in QIH group, indicating that oscillations may be less stable in the hypothermic state (Figs 1B, 2B and S2B). This variability could reflect a modest phase-resetting effect of temperature that depends on the time of day [30,31], consistent with previous reports in other heterothermic species, such as the bearded dragons [32]. In addition to the effect of hypothermia, the excitation of Q neurons could directly affect the phase of the SCN rhythms through the hypothalamic neuronal network (S1B Fig). Therefore, although our electrophysiological data strongly support that the circadian period remains close to 24 h during QIH, the precise calculation of Q_10_ was difficult.

The relationship between circadian clocks and hibernation has long been debated. In some species, photoperiodic cues detected by the circadian clock are critical for the seasonal onset of hibernation [33]. Melatonin synthesis, tightly regulated by the SCN, also plays a central role in photoperiodic adaptation. Moreover, the timing of torpor entry or arousal is sometimes circadian-regulated, as reported in bats and bears [34–36]. Interestingly, glucose uptake in the SCN of ground squirrels increases during torpor, suggesting that the SCN remains metabolically active [37]. A recent study indicated that oscillation with a period of multi-day modulates the circannual oscillation in golden hamster, which contributes to the timing of interval arousal bouts [38], raising the possibility that cold-resistant oscillations revealed in QIH are functionally relevant to the temporal organization of hibernation. By contrast, many rodent studies indicate a pause in circadian clock function during deep torpor: the expression of core clock genes in the SCN of European hamsters and Arctic ground squirrels was strongly attenuated or abolished [21,39]. Such discrepancies likely reflect species differences in body temperature regulation (e.g., bears remain ~34 °C, while small rodents fall below 10 °C) as well as differences in environmental cues. Importantly, studies in naturally hibernating animals cannot fully exclude the effect of zeitgeber signals, such as light or ambient temperature fluctuations, making it difficult to assess intrinsic circadian properties.

Here, using QIH under strictly controlled environmental conditions, we describe circadian dynamics with high resolution in a hypothermic state. Our findings show that oscillations in behavior, SCN neuronal activity, and peripheral PER2::LUC expression persist, albeit with reduced amplitude, even when body temperature remains at ~25 °C for extended periods. This contrasts sharply with in vitro slice cultures, where PER2::LUC expression rhythms were strongly damped in the SCN slices or reset at the same temperature in the kidney slices (Fig 5). However, the circadian amplitude at low temperature should be carefully interpreted here because the luciferase-based monitoring includes multiple temperature-sensitive processes such as luciferin metabolism, ATP production, and luciferase enzymatic activity. It is necessary to address whether the circadian oscillation persists or not by using other methods, such as direct quantification of mRNAs or proteins in vivo and vitro cultures. A recent study also demonstrated that intracellular Ca^2+^ concentration in the SCN slice cultures remains even at 22 °C, despite attenuation of *Per2* gene expression [29], implying oscillation of Ca^2+^ was possibly reserved in our in vitro study. It is also noted that the circadian dynamics in QIH mice examined in this study are influenced by not only the temperature changes but also the continuous excitation of Q neurons. To segregate these effects, further study using the warm-QIH model, in which the mouse is kept at high ambient temperature to prevent the decrease in body temperature, is needed.

Many studies on temperature compensation in mammalian cultures have compared relatively mild conditions (30–32 °C versus 37 °C) [6,16,17], since rhythmicity becomes difficult to monitor at lower temperatures due to damping. In contrast, we observed clear rhythmic patterns of SCN firing and peripheral PER2::LUC in living mice, even in the deep QIH state. Moreover, the period change during QIH was milder compared to that observed in cold-exposed slice cultures [29]. QIH represents an actively induced hypometabolic state rather than forced cold exposure in culture systems. Thus, we speculated that metabolism, such as glucose expenditure, can be different between QIH and in vitro cultures. The systemic signals in QIH, absent in culture, also possibly contribute to these in vivo versus in vitro differences. These may include metabolic feedback (e.g., redox oscillations), neuroendocrine factors such as melatonin or corticosterone, or autonomic nervous system inputs that stabilize cellular oscillators. Network-level coupling within the SCN may allow residual Ca²⁺ and electrical activity to maintain coherent rhythms even when transcriptional oscillations are weakened [40].

In conclusion, our study demonstrates that the mammalian circadian clock works with a period robustly temperature compensated in hypometabolic and hypothemic animals. This robustness is likely conferred by organism-level regulatory mechanisms that buffer cell-autonomous clocks against hypothermia. These findings address a long-standing question in circadian biology, and they provide a conceptual framework for understanding how clocks function during hibernation-like states. Moreover, by highlighting systemic contributions to circadian robustness, our results may inform translational applications of medical hypothermia, where maintaining circadian integrity could influence recovery and therapeutic outcomes.

## Materials and methods

### Animals

*Qrfp-iCre* mice have been described previously [24]. As:mPer2Luc (referred to as PER2::LUC) mice were obtained from the Jackson Laboratory (stock no. 006852) [27]. Male and female *Qrfp-iCre* heterozygotes with a C57BL/6J background were used for behavioral assessment and electrophysiological recording. *Qrfp-iCre* heterozygous and PER2::LUC homozygous mice were used for in vivo PER2::LUC recording. PER2::LUC homozygous mice were used for in vitro tissue cultures. Ten- to 20-week-old mice were used in this study. The mice were maintained under a 12h/12 h light/dark cycle (LD) or constant darkness (DD) with food and water provided ad libitum. They were in a temperature-(23 ± 1 °C) and humidity-controlled room or a thermal chamber (HC-100, SHICFACTORY, Japan), in which the temperature was controlled at 22 ± 1 °C. All experimental procedures were approved by the Animal Experiment and Use Committee of the University of Tsukuba (approval number: 25-077) or the OIST Animal Care and Use Committee (approval number: ACUP-2023-002), and were performed in accordance with the NIH guidelines.

### Virus preparation

AAVs were generated using the AAVpro Helper Free System (Cat #6232; Takara Bio Inc.). pHelper, pRC, and each pAAV vector were transfected into HEK293T cells using polyethylenimine (Cat#24765, Polyscience) according to a standard protocol. Three days after transfection, the virus was purified using an AAV extraction solution (Cat #6235; Takara Bio Inc.) according to the manufacturer’s protocol. The titer of virus was confirmed by real-time PCR. AAV10-Ef1a-DIO-hM3D(Gq)-mCherry; 1.0 × 10^13^ genome copies/ml, AAV10-Ef1a-DIO-mCherry; 1.0 × 10^13^ genome copies/ml.

### Surgery and QIH induction

For the induction of QIH, AAV10-Ef1a-DIO-mCherry (for control) or AAV10-EF1a-DIO-hM3D(Gq)-mCherry was bilaterally injected into the AVPe of *Qrfp-iCre* mice. The mice were anesthetized with 1.5%− 2% isoflurane inhalation solution (Viatris) for all procedures. Mice were positioned within a stereotaxic frame (David Kopf Instruments). The injection coordinates for AVPe were: AP, 0.5 mm; ML, ± 0.25 mm from bregma; DV, −5.3 mm from the skull surface. The injection volume at each site was 330 nl with a glass microcapillary syringe using an air pressure injector system (Picospritzer III, Parker). We waited 5 min before removing the syringe after injection. The mice were housed in their home cages for over 2 weeks before experiments (behavioral and PER2::LUC monitoring). During the recovery from surgery, the mice were entrained to LD 12:12h cycle. The mice were habituated to intraperitoneal injection of saline once or twice before the experiments. Mice were then intraperitoneally injected with 1 mg/kg of clozapine-*N*-oxide (CNO; Cat# ab141704, abcam) dissolved in saline. The viral expression was confirmed by immunohistochemistry after the experiments.

### Immunohistochemistry (IHC)

Animals were deeply anesthetized with isoflurane (Viatris), and then perfused transcardially with chilled phosphate-buffered saline (PBS), followed by 4% paraformaldehyde (PFA, Cat# 02890-45, Nacalai Tesque) in PBS. Brains were removed and postfixed overnight in 4% PFA at 4 °C and transferred to 20% sucrose (Cat# 196–00015, FUJIFILM Wako Pure Chemical Corporation) in PBS at 4 °C. After overnight incubation, the brains were frozen in liquid nitrogen in the gas phase. Coronal brain sections including AVPe, POA, SCN, or LH were prepared using a cryostat (Leica Biosystems). The serial brain sections were collected in PBS and incubated with blocking buffer (0.2 v/v% Triton X-100 [Cat# 807426, MP Biomedicals], 3% bovine albumin serum pH 5.2, [Cat# 01863-48, Nacalai Tesque]/PBS) for 30 mins at room temperature. The sections were incubated with primary antibody, goat anti-mCherry (1:1000; SICGEN Cat# AB0040-200, RRID: AB_2333092), in the blocking buffer at 4 °C overnight. The sections were rinsed two times for 10 mins each with PBS and once for 10 mins with blocking buffer, followed by incubation with a secondary antibody, Alexa Fluor 594 donkey anti-goat (1:1000; Thermo Fisher Scientific Cat# A11058, RRID: AB_2534105), at either room temperature for 3 h or 4 °C overnight. To detect the expression of c-Fos, c-Fos antibody (Cat#: EE4-RPCA-C-FOS-100, EnCor Biotechnology Inc.) and Alexa Fluor 488 donkey anti-rabbit (1:1000; Thermo Fisher Scientific, Cat#: A-21206, RRID: AB_2535792) were used. To confirm the position of the SCN, sections were counterstained with 1 mg/ml Ellstain DAPI solution (Cat# D523, Dojindo) diluted by PBS. After incubation, the sections were rinsed three times for 10 min each with PBS, mounted, and coverslipped. Images were captured under a microscope (Leica SP8, Leica Microsystems, or AxioZoom V16, Carl Zeizz).

### Locomotor and wheel-running activity recording

The mice receiving viral injection were housed individually in their home cages with infrared light sensors (Labdesign, Cat# ACT-1) or cages equipped with running wheels (Cat# RWC-15, Melquest). The cages were maintained in light-tight chambers illuminated with white light-emitting diode (LED; 100 lux). Mouse activities were recorded in 1-min bins (Clocklab, Actimetrics) in either LD or DD. Mice were first entrained to LD cycle and then transferred to DD. About one week after mice were transferred to DD, QIH was induced by 1 mg/kg CNO i.p. Mice were continuously kept in the DD after the QIH induction for at least 1 week. The activity onsets were determined by ClockLab Analysis version 6 (Actimetrics) for 7 days before the QIH induction and 6 days after the recovery from QIH, unless data were missing due to technical issues. In almost all cases, the recovery time was determined as 72 h after the QIH induction (CNO i.p.) for both control and QIH groups. However, in wheel-running activity measurement, some mice showed hypoactivity longer than 72 h. In this case, the recovery time was set to 96 h or 120 h after the QIH induction based on the activity level. The phase shift between the pre-QIH and post-QIH on the first day after the recovery was calculated. Positive values of phase shift mean phase advance, while negative values mean phase delay. The circadian period was calculated by fitting a line to onsets in ClockLab. The time of activity onset was defined as CT 12.

### Brain temperature recording from free-moving mice

Our SCN temperature measurement system was modeled after a previously published model used for the preoptic area [41]. Briefly, an ultra-thin thermocouple probe (Cat#IT-24P, Physitemp Instruments) was inserted into a cannula, which is usually used for intraventricular administration (Eicom, Japan). The edge of the thermocouple probe was exposed by 0.5 mm from the cannula and fixed to the cannula. A guide cannula was implanted into the mouse brain. The coordinates for implantation into the SCN were: AP, −0.46 mm; ML, 0.2 mm from bregma; DV, −5.2 mm from the skull surface with an angle of 15°. The dummy cannula was protected by a cap until the recording. The mice were housed in their home cages for over 2 weeks before experiments. During the recording of brain temperature from the free-moving mice, the dummy cannula was replaced with a cannula with a thermocouple probe. The temperature was recorded using THERMES-USB Temperature Data Acquisition (Physitemp Instruments) and DASY lab software (measX). The data was collected at 2 Hz.

### Body temperature recording

To evaluate the core body temperature, a thermal logger, iButton (DS1922L, Analog Devices) or NanoTag (VSMN210, Kissei Comtec), was implanted intraperitoneally in mice after the behavioral assessment. The core body temperature was recorded every 10 min for 2 weeks. The amplitude and period of body temperature rhythms were calculated by fitting the data to a sine curve. Three days before the CNO injection was used for pre-QIH (days 1–3). Three days after the recovery (day 9–11) was defined as post-QIH. The first peak time of body temperature during Pre- and Post-QIH periods was used for the calculation of phase shift, in which the period was assumed to be 24 h. To record core body temperature and brain temperature simultaneously, a thermo-sensor (TA11TA-F10, DSI) was implanted intraperitoneally in mice [25,42]. The core body temperature was recorded at 1 Hz.

### Electrophysiology recording

For electrophysiological recording, we used the microdrive with 8 independently adjustable tetrodes (14 nm diameter, nichrome wire, gold plated to the impedance of 200–300 kΩ). Another adjustable tetrode located outside of SCN served as a reference. The recording tetrodes were placed in SCN (the same coordinate described above).

All animals were single-housed prior to surgery. During the surgery, mice were anesthetized with MMB (a mixture of midazolam, medetomidine, and butorphanol tartrate at concentrations of 4.0 mg/kg, 0.3 mg/kg, and 5.0 mg/kg, respectively; intraperitoneally (i.p.) injected at the dose of 10 mL/kg). After the virus injection to AVPe, the microdrive was implanted at the target location. One of the stainless screws attached to the skull was used as an electrical ground for extracellular recording.

After surgery, the health conditions of the animals were carefully monitored every day. After recovery, the depth of the tetrodes was slowly and manually adjusted until a stable recording of SCN units was achieved. To confirm SCN units, mice were placed in a familiar box with food and water provided, and their spike activity was recorded 48 h at the LD condition and another 48 h at the DD condition (10 min recording for each hour, repeated 48 times for LD and DD). Only units that showed the circadian fluctuations during both LD and DD conditions were used for the experiments.

All data were acquired using a 32-channel Digital Lynx SX acquisition system (Neuralynx). Spike waveforms were filtered between 0.6 and 6 kHz and those above a peak threshold of 50 μV were time-stamped and digitized at 32,556 Hz. Spikes were manually sorted using SpikeSort3D software (Neuralynx). Briefly, spike data from all the recording sessions of 48 h were first concatenated for spike sorting. The waveform variables (peak amplitudes and their energies) of all spikes recorded from a combination of three recording channels were projected to 3D space, and putative units were manually clustered. This procedure was repeated for all combinations of channels. Clusters with >0.5% spikes having inter-spike-interval of <2 ms were excluded. The firing rates were standardized to compare with that from other electrodes or animals. The standardized firing rate was fitting to a sine curve to calculate the period and amplitude.

After all recording sessions, mice were anesthetized for electrolytic lesions to mark tetrode locations and then transcardially perfused. Following 24 h of post-fixation with 4% PFA (paraformaldehyde), brains were sectioned (50 μm thickness) to confirm tetrode locations in SCN.

### In vivo recording of PER2::LUC

The *Qrfp-iCre* heterozygous/*PER2::LUC* homozygous mice receiving AAV10-Ef1a-DIO-hM3D(Gq)-mCherry were used. An osmotic pump (Cat#2001, Alzet) filled with 200 µl 150 mM D-luciferin potassium salt (Cat# 126–05116, FUJIFILM Wako Pure Chemical Corporation) dissolved in water was implanted intraperitoneally in the mice. The hair around the back of the mouse was shaved thoroughly by a shaver. The infusion rate was 1 µl per hour, and the infusion persisted for at least 1 week. After the implantation, mice were placed in the Multi-function In vivo Imaging System (MIIS, Molecular Devices) and provided with gel water (Cat#70-01-5022, ClearH_2_O) and pellet food. The bioluminescence was detected every 10 min with a CCD camera (EM gain: 1,000, binning: 2x2, exposure time: 5 s) in constant dark condition. Two days after the recording started, QIH was induced by the injection of 1 mg/kg CNO and put back in the imaging chamber. The bioluminescence signal intensity was analyzed by ImageJ software. After detrending data by subtracting the values of 24-h moving average, a sine curve was fitted to the detrended data to determine the phase, period, and amplitude.

### Tissue slice cultures and in vitro PER2::LUC recording

The PER2::LUC homozygous mice were sacrificed by spinal dislocation during the light phase. For the SCN culture, the brains were rapidly removed and placed on ice in 1 × Hanks’ balanced salt solution (HBSS, Cat# 14025–092, Thermo Fisher Scientific) on ice. 150 µm coronal sections including the SCN were made with the vibratome (VT1200, Leica Biosystems). A small section of ~2 mm^2^ including the SCN, was cut from each slice with a razor in chilled HBSS. For kidney cultures, 100–150 µm sections were prepared with the vibratome in HBSS. One section was then divided into two pieces using a razor. The each slice was then placed on a Millicell Cell Culture Inserts (Cat# PICM0RG50, Merck KGaA) in a 35 mm tissue culture dish (Cat# 3000–035, IWAKI AGC Techno Glass) with 1.5 mL of low glucose Dulbecco’s modified Eagle’s medium (Cat# D2902, Merck KGaA) containing 10 mM HEPES (pH 7.0), 3.5 mg/ml D-glucose, 0.1 mM D-luciferin potassium salt (Cat# 126–05116, FUJIFILM Wako Pure Chemical Corporation), 2% B-27 supplement (Cat# 17504–044, Thermo Fisher Scientific), 35 mg·L^−1^ NaHCO3 (Cat# 197–01302, FUJIFILM Wako Pure), and 1% penicillin–streptomycin (10,000 units/ml penicillin and 10,000 mg/ml streptomycin; Cat# 15140–122, Thermo Fisher Scientific). Cultures were maintained at 37 °C or 25 °C, and bioluminescence was recorded for 1-min every 10 min with luminometer Kronos Dio (AB-2550, ATTO). Bioluminescence signals were detrended by subtracting 24-h moving average. The amplitude and period of bioluminescence rhythms were calculated by fitting the data to a sine curve.

### Statistical analysis

No statistical methods were used to determine the sample size. The experiments were randomized, and the investigators were not blinded to the allocation during the experiments. All statistical analyses were performed using GraphPad Prism 10. Differences were considered significant at *p < 0.05, **p < 0.01, and ***p < 0.001. The statistical test results and sample numbers were described in each figure legend and S1 Data.

## Supporting information

S1 Fig hM3Dq-mCherry expression in Q neurons. (**A**) Schematic drawings along the rostral–caudal axis of the preoptic hypothalamic region, with the three middle drawings (approximately bregma 0.50–0.26 mm) indicating the center of the AVPe region (left). Corresponding representative fluorescent images show the extent of virally delivered, Cre-dependent hM3Dq-mCherry expression in a Qrfp-iCre mouse (right). The right panels show representative microscopic images corresponding to the boxed areas indicated in the atlas schematics on the left. AAV was injected at the target coordinate indicated in the figure. The somata of mCherry-positive cells are confined in the area between bregma 0.50 mm and 0.22 mm, with no detectable somata observed caudal to bregma 0.14 mm. Scale bars, 200 µm. The distances from the bregma were determined using the brain atlas [43] and are shown in the images. (**B**) Representative images of axonal projection of hM3Dq-mCherry-positive neurons. Axonal projections are observed in the vicinity of the SCN but are sparse within the SCN. Bottom, axonal projections are observed in the LHA and DMH, whereas no somata are detected in these regions. Scale bars, 200 µm. Abbreviations: AVPe, antero-ventral periventricular nucleus; 3V, third ventricle; SCN, suprachiasmatic nucleus; LHA, lateral hypothalamic area; DMH, dorsomedial hypothalamic nucleus.
(TIF)

S2 Fig Effect of QIH on mouse wheel-running behavior. (**A**) Actograms of wheel-running activity for mice in the QIH condition. The timing of start of constant darkness (DD) and QIH induction was indicated by the arrowheads. The timing of CNO injection was presented as a star in the actogram. (**B**) Phase shift of the wheel-running activity rhythms between the pre-QIH and post-QIH period. The time in the graph was the induction time of QIH. Data were shown as means with individual plots and SD (Control = 17, QIH *n* = 20, Control versus QIH: *p* = 0.0859 by Welch’s *t* test, p = 0.0011 by F test). When the mice showed insufficient QIH (minimum body temperature of above 27 °C), the sample was excluded from analysis. (**C**) Correlation between the phase shift and the circadian time (CT) at the CNO administration. When the mice showed insufficient QIH (minimum body temperature of above 27 °C), the sample was excluded from analysis (QIH: *n* = 20, *p* = 0.4561, r = –0.1767 by Pearson’s correlation). (**D**) Correlation between the phase shift and the minimum temperature after the CNO administration. It includes data from all QIH mice expressing hM3Dq-mCherry regardless of the degree of minimum body temperature (QIH ≦ 27 °C: *n* = 20, QIH > 27 °C: *n* = 12) and control mice expressing mCherry (*n* = 17). *p* = 0.0352, r = –0.3016 by Pearson’s correlation. The data underlying this Figure can be found in S1 Data.
(TIF)

S3 Fig Brain temperature recording from free-moving mice. (**A**) Scheme showing the methodology to measure abdominal body (core) and brain temperature near the SCN in freely moving mice under QIH. A guide cannula was positioned above the SCN, through which a thermocouple probe was inserted during the recording. (**B**) An example of post-hoc histological micrograph. (Top) Expression of hM3Dq-mCherry in the AVPe. (Bottom) Placement of the thermocouple probe above the SCN. The position of the SCN was confirmed by DAPI stain. Scale bar, 200 µm. (**C**) Infrared thermography shows stable trunk temperature before QIH and during QIH. (**D**) Representative traces of locomotor activity and temperature (brain SCN, red; abdominal core, blue) from a single mouse showing before and after QIH. QIH was induced at 6 h by intraperitoneal injection of CNO. The bottom panel shows an excerpt from 5–10 h. Simultaneous temperature recordings were obtained independently from three mice, yielding similar results. Data from these three mice are found in S1 Data. (**E**) Plots show the mean ± SD of brain and core temperatures across three mice. Each panel illustrates a 2-h excerpt taken from three phases: the induction period, the time when the temperature first reached its minimum, and deep QIH, during which brain and core temperatures were nearly indistinguishable. (**F)** The differences between core and brain temperatures (ΔT = Tcore − Tbrain) were plotted for the three time windows. The data underlying this Figure can be found in S1 Data.
(TIF)

S1 Data All data shown in this study. (XLSX)

## Abbreviations

AVPe: antero-ventral periventricular nucleus
CNO: clozapine-*N*-oxide
CT: circadian time
DREADD: Designer Receptors Exclusively Activated by Designer Drugs
IHC: Immunohistochemistry
PBS: phosphate-buffered saline
PER: PERIOD
QIH: Q neuron–induced hypometabolic and hypothermic state
QRFP: pyroglutamylated RFamide peptide
SCN: suprachiasmatic nucleus.
