# Supplementary figures and images for: Circadian rhythms remain temperature compensated during a Q neuron–induced hibernation-like state in mice
Source: PLoS Biol. 2026 Apr 15;24(4):e3003475. doi: 10.1371/journal.pbio.3003475 (PMC13099087; doi:10.1371/journal.pbio.3003475)

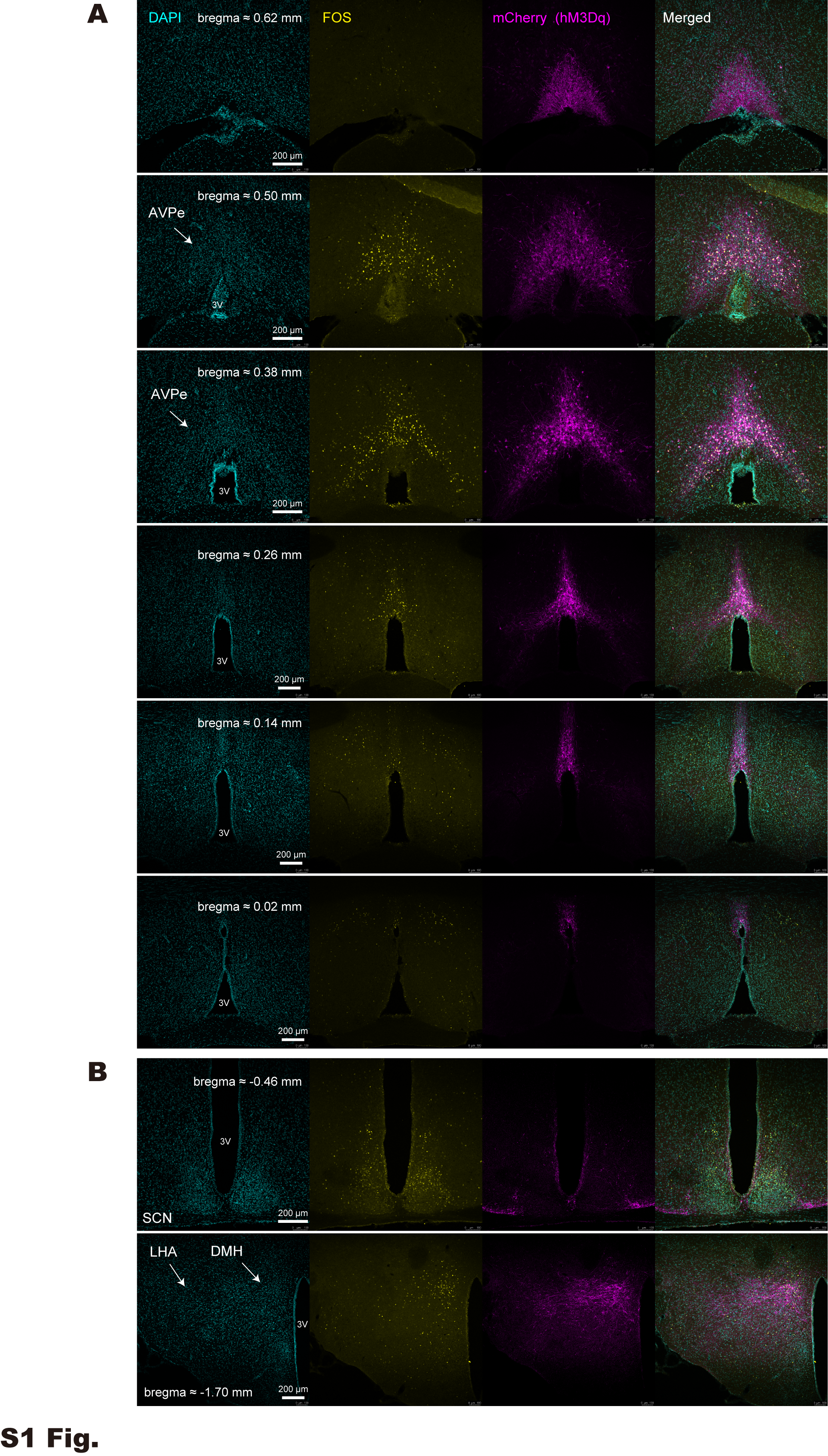

Supplement: S1 Fig — (A) Schematic drawings along the rostral–caudal axis of the preoptic hypothalamic region, with the three middle drawings (approximately bregma 0.50–0.26 mm) indicating the center of the AVPe region (left). Corresponding representative fluorescent images show the extent of virally delivered, Cre-dependent hM3Dq-mCherry expression in a Qrfp-iCre mouse (right). The right panels show representative microscopic images corresponding to the boxed areas indicated in the atlas schematics on the left. AAV was injected at the target coordinate indicated in the figure. The somata of mCherry-positive cells are confined in the area between bregma 0.50 mm and 0.22 mm, with no detectable somata observed caudal to bregma 0.14 mm. Scale bars, 200 µm. The distances from the bregma were determined using the brain atlas [43] and are shown in the images. (B) Representative images of axonal projection of hM3Dq-mCherry-positive neurons. Axonal projections are observed in the vicinity of the SCN but are sparse within the SCN. Bottom, axonal projections are observed in the LHA and DMH, whereas no somata are detected in these regions. Scale bars, 200 µm. Abbreviations: AVPe, antero-ventral periventricular nucleus; 3V, third ventricle; SCN, suprachiasmatic nucleus; LHA, lateral hypothalamic area; DMH, dorsomedial hypothalamic nucleus. (TIF) [file pbio.3003475.s001.tif]

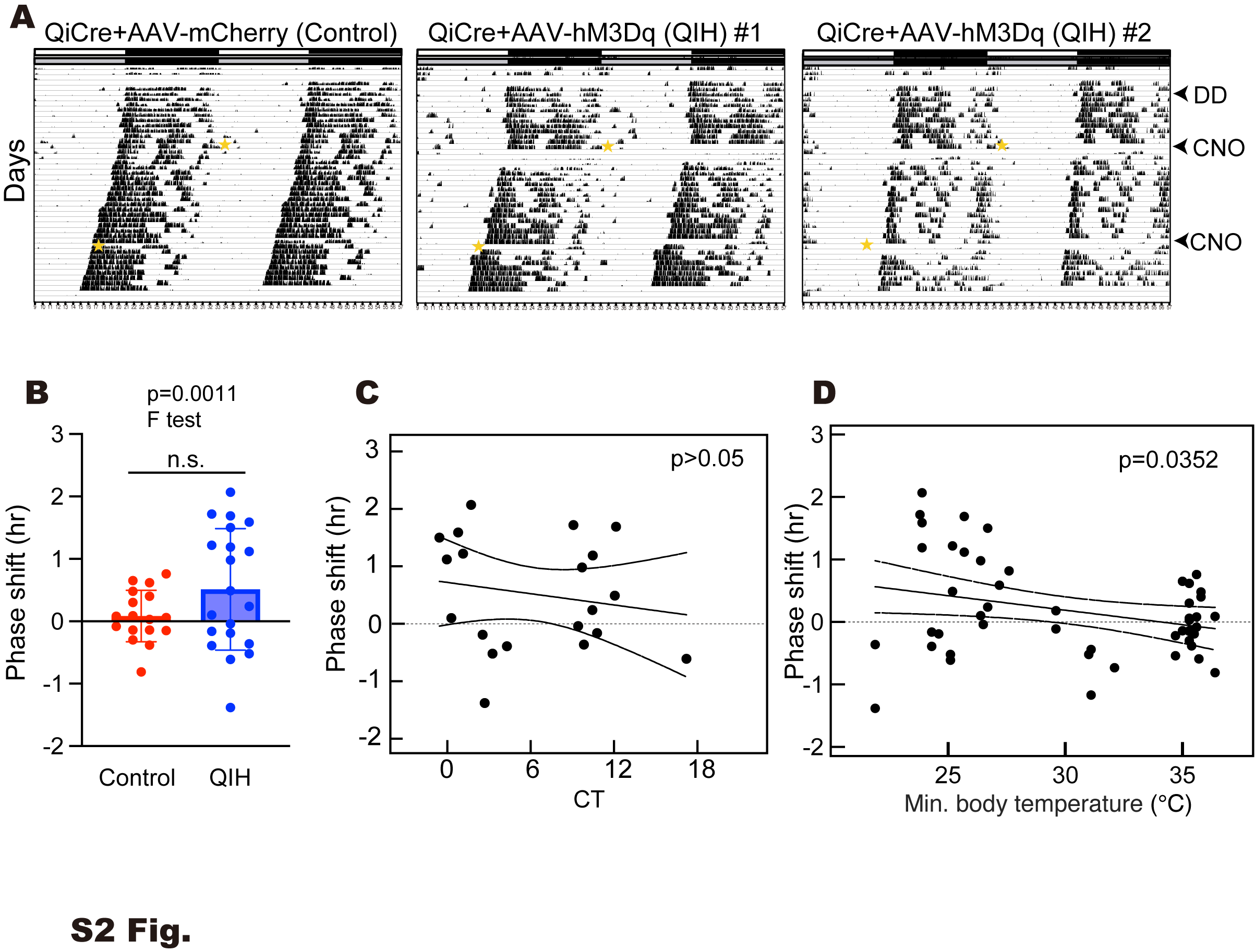

Supplement: S2 Fig — (A) Actograms of wheel-running activity for mice in the QIH condition. The timing of start of constant darkness (DD) and QIH induction was indicated by the arrowheads. The timing of CNO injection was presented as a star in the actogram. (B) Phase shift of the wheel-running activity rhythms between the pre-QIH and post-QIH period. The time in the graph was the induction time of QIH. Data were shown as means with individual plots and SD (Control = 17, QIH n = 20, Control versus QIH: p = 0.0859 by Welch’s t test, p = 0.0011 by F test). When the mice showed insufficient QIH (minimum body temperature of above 27 °C), the sample was excluded from analysis. (C) Correlation between the phase shift and the circadian time (CT) at the CNO administration. When the mice showed insufficient QIH (minimum body temperature of above 27 °C), the sample was excluded from analysis (QIH: n = 20, p = 0.4561, r = –0.1767 by Pearson’s correlation). (D) Correlation between the phase shift and the minimum temperature after the CNO administration. It includes data from all QIH mice expressing hM3Dq-mCherry regardless of the degree of minimum body temperature (QIH ≦ 27 °C: n = 20, QIH > 27 °C: n = 12) and control mice expressing mCherry (n = 17). p = 0.0352, r = –0.3016 by Pearson’s correlation. The data underlying this Figure can be found in S1 Data. (TIF) [file pbio.3003475.s002.tif]

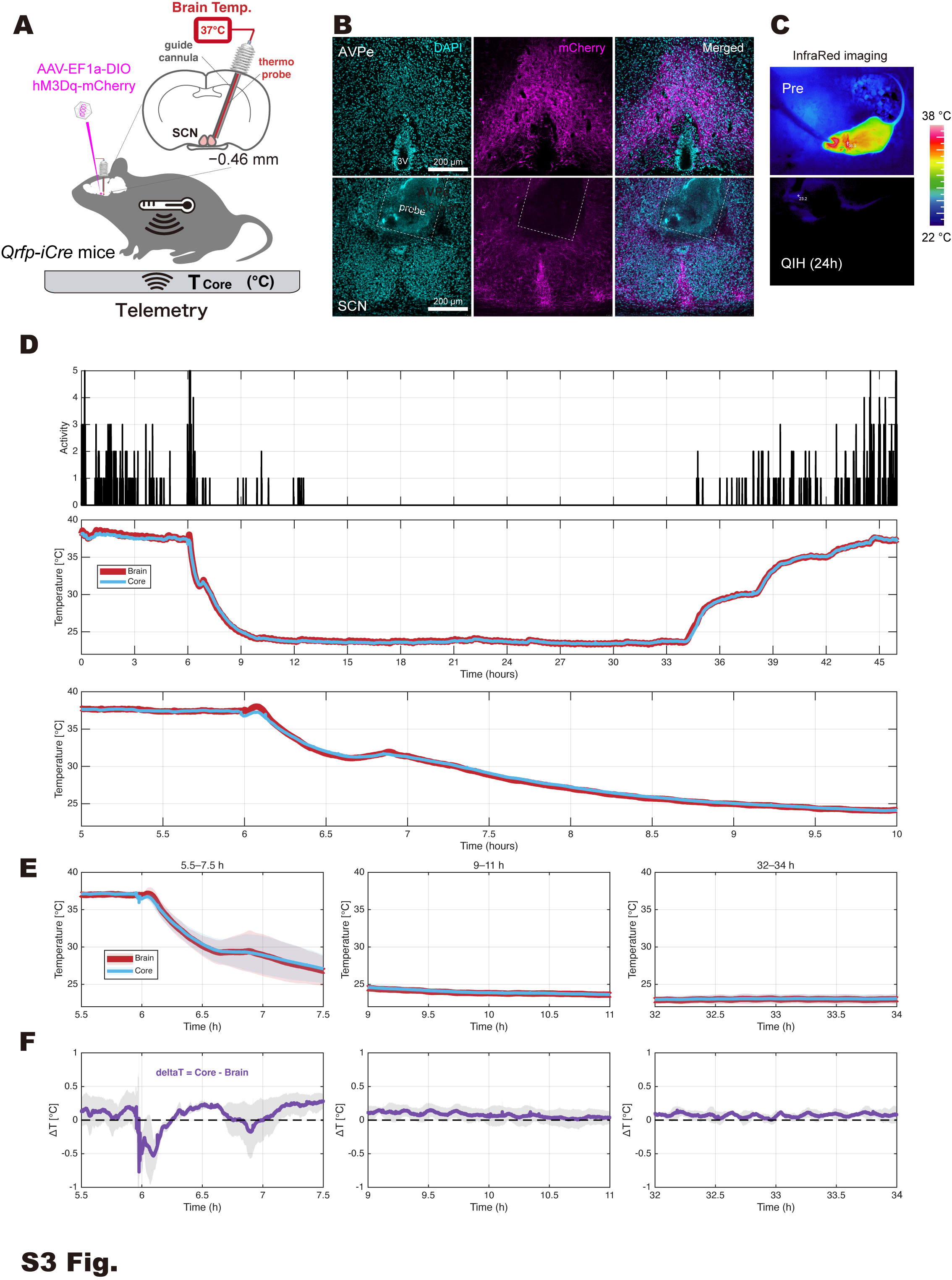

Supplement: S3 Fig — (A) Scheme showing the methodology to measure abdominal body (core) and brain temperature near the SCN in freely moving mice under QIH. A guide cannula was positioned above the SCN, through which a thermocouple probe was inserted during the recording. (B) An example of post-hoc histological micrograph. (Top) Expression of hM3Dq-mCherry in the AVPe. (Bottom) Placement of the thermocouple probe above the SCN. The position of the SCN was confirmed by DAPI stain. Scale bar, 200 µm. (C) Infrared thermography shows stable trunk temperature before QIH and during QIH. (D) Representative traces of locomotor activity and temperature (brain SCN, red; abdominal core, blue) from a single mouse showing before and after QIH. QIH was induced at 6 h by intraperitoneal injection of CNO. The bottom panel shows an excerpt from 5–10 h. Simultaneous temperature recordings were obtained independently from three mice, yielding similar results. Data from these three mice are found in S1 Data. (E) Plots show the mean ± SD of brain and core temperatures across three mice. Each panel illustrates a 2-h excerpt taken from three phases: the induction period, the time when the temperature first reached its minimum, and deep QIH, during which brain and core temperatures were nearly indistinguishable. (F) The differences between core and brain temperatures (ΔT = Tcore − Tbrain) were plotted for the three time windows. The data underlying this Figure can be found in S1 Data. (TIF) [file pbio.3003475.s003.tif]
